# An Overview of Artificial Intelligence in Gynaecological Pathology Diagnostics

**DOI:** 10.3390/cancers17081343

**Published:** 2025-04-16

**Authors:** Anna Joshua, Katie E. Allen, Nicolas M. Orsi

**Affiliations:** 1 Christian Medical College, Vellore 632004, Tamil Nadu, India; annajoshua0412@gmail.com; 2 Women’s Health Research Group, Leeds Institute of Cancer & Pathology, Wellcome Trust Brenner Building, St James’s University Hospital, Beckett Street, Leeds LS9 7TF, UK; umkem@leeds.ac.uk

**Keywords:** artificial intelligence, gynaecological malignancies, pathology, ovarian cancer, endometrial cancer, cervical cancer, vulval cancer

## Abstract

**Background**: The advent of artificial intelligence (AI) has revolutionised many fields in healthcare. More recently, it has garnered interest in terms of its potential applications in histopathology, where algorithms are increasingly being explored as adjunct technologies that can support pathologists in diagnosis, molecular typing and prognostication. While many research endeavours have focused on solid tumours, gynaecological malignancies have nevertheless been relatively overlooked. The aim of this review was therefore to provide a summary of the status quo in the field of AI in gynaecological pathology by encompassing malignancies throughout the entirety of the female reproductive tract rather than focusing on individual cancers. **Methods**: This narrative/scoping review explores the potential application of AI in whole slide image analysis in gynaecological histopathology, drawing on both findings from the research setting (where such technologies largely remain confined), and highlights any findings and/or applications identified and developed in other cancers that could be translated to this arena. **Results**: A particular focus is given to ovarian, endometrial, cervical and vulval/vaginal tumours. This review discusses different algorithms, their performance and potential applications. **Conclusions**: The effective application of AI tools is only possible through multidisciplinary co-operation and training.

## Simple Summary

Artificial intelligence (AI) is contributing to healthcare, including histopathology, by providing tools for diagnosis, molecular typing and prognostication. Gynaecological tumours are a relatively under-researched area in this setting. This review focuses on how AI could be potentially applied to the histopathological imaging of cancers of the reproductive tract, mainly ovarian, endometrial, cervical and vulval/vaginal tumours. It also explores whether AI in other cancers can be incorporated to improve outcomes of gynaecological cancers. It emphasises the need for a multidisciplinary approach required for the effective implantation and functioning of these tools.

## 1. Introduction

The use of digital pathology in routine diagnostic histopathology has greatly increased over the past decade, wherein pathologists increasingly have the opportunity to review whole slide images (WSIs) on high-resolution computer screens in digitised clinical centres [1]. There is a growing body of evidence to suggest that reporting from WSIs has made case reviews faster, more efficient and more focused [2,3]. The corollary of the digitisation of histopathology services has been the creation of large clinical and academic WSI repositories with the potential to form the developmental backbone for new adjunct artificial intelligence (AI) platforms that aim to improve diagnostics and prognostication as well as molecular subtyping and genomic profiling [4]. In turn, these solutions promise to lay the foundations for research and drug discovery programmes and the emergence of personalised medicine. Access to analytical platforms should also prove invaluable in light of the increasing global shortage of pathologists [5], which has been partly driven by the rising diagnostic service demand and the parallel increasing complexity of diagnostic modalities (e.g., immunohistochemistry and next-generation sequencing). 

This vulnerability in histopathology diagnostic services is particularly pronounced in developing economies, where the lack of pathologists is compounded by the limited availability and accessibility of ancillary testing, which commonly results in delayed diagnoses, incomplete diagnostic profiling and difficulties in accessing both targeted and timely cancer management. Given that the clinical utility of such solutions depends in part on healthcare diagnostic service digital infrastructures, the provision of such resources could enable AI platforms to help overcome the scarcity of pathologists and affiliated resources in these environments [6]. In this regard, the adoption and integration of diagnostic AI into digital pathology workflows has been purported to offer several potential benefits, including accelerating diagnoses (by decreasing case turnaround times), improving patient safety (by providing an objective second opinion), streamlining workflows (by incorporating flexible and remote working), overcoming workforce constraints (by decreasing workload) and improving service quality [7]. 

However, AI has other potential benefits to offer in the diagnostic arena. Consistency in histopathological evaluation is recognised to be affected by occasionally poor intra-pathologist consensus owing to differences in visual assessment and in interpretation of clinical data [8]. This could be improved with the help of AI tools, which, when appropriately deployed, can be both objective and consistent in their provision of an independent diagnostic opinion. This has been suggested to be of particular value in less specialised environments, such as outwith tertiary referral centres [9].

One of the major challenges with the adoption of digital pathology and allied AI platforms is its acceptance among clinical histopathologists. Whilst the concept of using diagnostic adjunct solutions may have initially been met with scepticism [10], a recent study reported an overall positive response of pathologists in accepting the use of AI in diagnostic pathology on the basis that its implementation could increase reporting efficiency and decrease errors [11]. Nevertheless, a substantial proportion of pathologists believe that a degree of training is necessary to underpin its fruitful application.

The use of AI in the wider oncology context has expanded over the past few years, ranging from its possible use in cancer genomics (such as identifying genomic alterations that may not be recognised by molecular panels) [12] through to radiological imaging and diagnostics [13]. From a research standpoint, the use of AI has made significant strides in multiple solid malignancies, including lung (chest radiography interpretation) [14] and gastric cancers (WSI evaluation) [15] in terms of both diagnostics and prognostication. Furthermore, Paige Prostate, an automated prostate cancer detection system using WSIs, has shown significant clinical effectiveness and achieved Food and Drug Administration (FDA) approval [16]. However, a detailed exploration of the potential applications and relative merits of AI in the gynaecological malignancy setting remains wanting. Therein, ovarian cancers account for the lion’s share of studies, which have focussed primarily on the use of deep learning (DL) in diagnostics [17,18]. The next most investigated area is claimed by endometrial cancer [19,20] and cervical screening, with other less common gynaecological malignancies largely remaining overlooked. This narrative/scoping review, therefore, aims to provide a timely update on the potential role of AI in the histopathological assessment of gynaecological cancers by encompassing malignancies throughout the entirety of the female reproductive tract and highlight any findings and/or applications identified and developed in other cancers that could be translated to this setting.

## 2. Methods

The literature supporting this review was based on extensive searches of PubMed and Embase using search strings encompassing the malignancies of interest (“endometrial cancer”, “ovarian cancer”, “cervical cancer”, “vulval cancer” and “vaginal cancer”) in association with “artificial intelligence”. The specific search strings used were (((“Artificial Intelligence” [Mesh]) AND “Ovarian Neoplasms” [Majr])) AND (pathology [Title/Abstract]), (endometrial cancer [Title/Abstract]) AND (“artificial intelligence” [Mesh]), (cervical cancer [Title/Abstract]) AND (“artificial intelligence” [Mesh]), (vulval cancer [Title/Abstract]) AND (“artificial intelligence” [Mesh]). Further refinements thereafter were introduced to exclude studies that focused on solely radiology, clinical metadata and/or genomic/transcriptomic profiling to maintain a stronger focus on digital histopathology. Further targeted searches were then applied using terminology to address lacunae in research studies in the gynaecological setting by applying searches to related (from a histomorphological perspective) clinical entities (“malignant melanoma”, “squamous cell carcinoma” and “squamous cell cancer”) in which AI has been applied to inform areas of possible future development.

## 3. Ovarian Cancer

Over 300,000 new cases of ovarian cancer were diagnosed globally in 2022 [21], with an associated mortality rate of just over 200,000. A significant contributor to the poor outcomes which characterise this disease is its propensity for late detection and diagnosis and the commonplace development of resistance to platinum-based chemotherapy [22]. Epithelial ovarian cancers account for most of the malignant ovarian tumours [23], with germ cell tumours and sex-cord tumours combined comprising the remaining 10% [24].

While ovarian masses are evaluated based on risk factors, clinical findings, imaging and tumour markers, the diagnosis is finalised histologically. Increasingly, cases are being reported digitally from WSIs, wherein the potential benefits of adjunct AI platforms could be brought to bear. In this regard, the possibility of automated histological classification of ovarian cancer with the help of a computer-aided diagnosis system has been explored by several research groups and shows great promise [25,26]. In earlier iterations, WSIs were used with a contextual model for the histological classification of ovarian cancer [27], and demonstrated good concordance with histopathologist-based diagnoses. In a 2022 study, four different DL models for ovarian cancer classification were developed using 948 WSIs. Of these, a one-stage transfer learning algorithm, which classified WSIs to one out of five morphological carcinoma subtypes, was found to be most efficient [28]. Furthermore, the possibility of integrating DL algorithms with multiphoton microscopy to analyse images of unstained tissue have also been explored in mouse models from ovarian and upper reproductive tract tissue [29]. By training neural networks on these images, researchers have been able to distinguish healthy tissue from serous carcinoma, highlighting the potential merit of such adjunct diagnostic platforms.

BRCA 1/2 genes—whose proteins orchestrate homologous recombination-dependent DNA repair—can be mutated in ovarian cancer, typically with a frequency of 25.7% in high-grade serous carcinoma [30]. Other instances of homologous recombination deficiency (HRD) phenotype are, to a lesser degree, also attributable to mutations in RAD51. Assessment of HRD is a keystone for informing treatment given that tumours displaying HRD exhibit heightened sensitivity to platinum-based chemotherapy and poly-ADP-ribose polymerase inhibitor (PARPi) combination therapy [31]. In this regard, Bourgade and colleagues described a novel approach for identifying BRCA mutations with the help of Convolutional Neural Networks (CNNs) and tumour segmentation from WSI of high-grade serous ovarian cancers [32], an approach which greatly reduced manual annotation times. This underscores the significant potential for improving diagnostic accuracy and personalised treatment strategies for patients with high-grade ovarian cancer harbouring BRCA mutations. Following its approval by the FDA and the European Medicines Agency (EMA), PARPi therapy has gained significant traction in clinical practice. However, its effectiveness has been constrained by challenges in accurately identifying HRD status. Although several genetic tests to detect HRD are available [33,34,35], no universal gold standard exists. In this regard, a single blinded study reported the use of an AI model to predict HRD status from H&E-stained WSIs alone, with a remarkable 99.3% accuracy [36], underscoring the fact that AI models could offer a promising alternative to determining HRD status and patient stratification, and overcoming the limitations of current testing modalities, including the relatively high failure rates and prolonged time required to obtain results.

Given the high cost and variable long-term efficacy of ovarian cancer treatment, predicting treatment response is an unattended requirement which has been investigated with the help of AI. For example, Wang and colleagues were able to predict the efficacy of bevacizumab therapy by analysing WSIs using a DL-based approach [37]. Similarly, a CNN-based model used WSIs to predict the impact of platinum-based chemotherapy on patients with high-grade ovarian cancer with a specificity of 91% and a sensitivity of 73% [38]. This was performed by associating tumour morphology to patient outcomes and digital biomarkers and quantified in terms of progression-free survival. This demonstrates the use of DL in targeted treatment by means of patient stratification and a possible prevention of resource wastage in both potentially ineffective therapy as well as over-treatment. Furthermore, a more recent study used such a model to identify morphological tumour regions with distinct transcriptional profiles by using spatial transcriptomics. This approach confirmed that these discrete regions had unique transcriptional signatures, which were more predictive of outcome than other background tumour regions [39]. Interestingly, the authors reported that the proto-oncogene JUN (which encodes the transcription factor c-Jun and is a central hub in the protein–protein interactions of tumours that recur rapidly after platinum-based treatment) was exclusively upregulated in these AI-detected areas.

AI models have shown a greater accuracy in predicting prognosis and survival rates as compared to traditional algorithms [40]. Employing DL techniques, Yang et al. developed a comprehensive index using H&E-stained WSIs, the Ovarian Cancer Digital Pathology Index (OCDPI), which predicts prognosis associated with adjuvant therapy. Patients were stratified into high and low OCDPI groups, and a significant association between the OCDPI and overall survival was demonstrated [41]. Prognosis was also assessed by Wu and colleagues by using deep learning models applied to ovarian cancer WSIs, wherein patients with a lower score showed better survival. Furthermore, it was found that a risk score had a better predictability of survival outcome with the HRD subgroup [42].

## 4. Endometrial Cancer

Endometrial cancer is the sixth commonest cancer occurring in women, with an annual incidence of over 400,000 cases worldwide and a mortality of around 97,000 [43]. It is also the commonest gynaecological malignancy in the developed world [44]. These cancers are classified based on their histology and hormone receptor expression. Moreover, the recent molecular classification of serous and endometrioid endometrial cancers categorises endometrial cancer into the following molecular subtypes: polymerase ε (POLE) mutated, mismatch repair-deficient (MMRd), p53 abnormal (p53abn) and no specific molecular type (NSMP) [45,46]. This classification is prognostically meaningful, especially in high-risk cases. POLE mutations have the best prognosis, while subtypes with p53 mutations have the worst. In addition to identifying invasive malignancy, there is a need to diagnose endometrial hyperplasia, the disordered proliferation of endometrial tissue with an altered gland-to-stroma ratio typically resulting from increased levels of unopposed oestrogen. The WHO [47] classifies endometrial hyperplasia as without atypia, a benign condition, and with atypia, a precancerous lesion which can progress to endometrial cancer. In this wider setting, endometrial biopsies are key to diagnosis.

During diagnosis using WSIs, pathologists face the task of differentiating benign from atypical or malignant tissue on slides, a process which can potentially be facilitated by using trained AI models [48,49,50]. Using 467 H&E stained endometrial specimen WSIs, Zhao and colleagues developed a CNN to diagnose endometrial hyperplasia with an accuracy of over 97% [49], which was further externally validated with an accuracy of over 95%. In another study, a DL model was developed to identify endometrial cancer from WSI patches—significantly, a specificity of 83.7% was achieved when specimens were assessed prospectively. This model was able to identify subtle areas on slides, thus providing a second opinion, prompting pathologists to revisit the case in the case of diagnostic discordance [20]. However, this work also highlights the major disadvantage of time-consuming annotation in developing and validating such WSI models. To address this challenge, in their later study a weakly supervised clustering-constrained attention-based multiple instance learning (CLAM) approach developed by Lu et al. [51] was utilised [52]. They demonstrated an area under the receiver operating curve (AUROC) of 95.19%, a 4.41% enhancement in accuracy in classifying endometrial tissue compared to using standard multiple instance learning (MIL).

EndoNet is another AI model that has been developed to classify endometrial cancers from hysterectomy specimen WSIs. EndoNet uses CNNs for extracting histological features and a vision transformer for aggregating these features and classifying WSIs into low- (endometrioid grades 1 and 2) and high-grade (endometrioid grade 3, uterine serous carcinoma or carcinosarcoma) categories with sustained performance (AUROC 0.86 on an external test set of images). The value of this solution is in its potential to support pathologists in grading tumours with greater consistency [53].

Moreover, there have been recent attempts to combine morphological classification with molecular typing in endometrial cancer. Im4MEC is such a DL model developed and tested using patient data (H&E-stained WSIs) from the Post-Operative Radiation Therapy in Endometrial Cancer (PORTEC) trial. Therein, researchers combined a self-supervised learning model with an attention-based classification model to interpret the data and were able to establish morphomolecular correlates and elaborate on intra-class heterogeneity [54]. Efforts have also been made to integrate AI in molecular tumour profiling, including the identification of functional mutations including their pathogenicity from cancer genome data [55,56]. Together, these algorithms could be used to predict the genomic profile of individual tumours and, in turn, contribute to informing treatment decisions (e.g., choice of targeted therapy), thereby potentially improving treatment outcomes [57]. However, these approaches could form part of a future solution incorporating WSI as part of multimodal diagnostic and prognostic platforms. In this respect, *Panoptes*, a CNN-based AI tool that identifies endometrial cancer molecular subtypes [58], uses a multi-resolution approach for H&E images, with 2.5×, 5× and 10× magnifications yielding three-tile grids. It classified histological subtypes with an AUROC of 0.969. Each slide was analysed in under four minutes, a turnaround time which could prove helpful to live reporting pathologists. This tool could also identify molecular patterns that may not be identifiable through visual assessment by a histopathologist, such as characteristics associated with driver mutations, thereby contributing to more precise diagnoses and the development of personalised treatment plans.

The conceptual move towards personalised therapy for women with endometrial cancer has also involved the image analysis of tertiary lymphoid structures (TLSs). Together with B cell infiltration into tumours, TLSs have been shown to be associated with a more favourable prognosis in endometrial cancer, putatively through their contribution to an intratumoural immunity amplification loop believed to increase tumour sensitivity to immunotherapy [59,60]. In this regard, Suzuki and colleagues developed an AI model which both detected TLSs and enabled the determination of their spatial locations in endometrial cancer WSIs [61]. Combined with molecular subtyping, TLS identification and positioning was predictive of both progression-free survival and response to immune checkpoint inhibitors. Given the recent incorporation of immunotherapy in the treatment of endometrial cancer, such platforms provide tantalising early signs of the potential value of AI in facilitating the implementation of personalised therapy in this setting.

As highlighted, although surgery is central to endometrial cancer management, targeted therapy and immunotherapy are promising new treatment modalities [62,63,64]. Tumour molecular profiles and clinicopathological factors (e.g., staging) contribute to prognosis of endometrial malignancies. Among the latter, the identification of lymph node metastasis stands out as being particularly significant. DL has been used to predict the probability of lymph node metastasis based on perioperative H&E imaging with WSIs from biopsy specimens [65]. The prediction achieved an AUC of 0.938 and 0.77 in internal and external cohorts, respectively. Furthermore, the heat maps generated from the specimens helped to visualise the involvement of different WSI regions for lymph node metastasis. Overall, this could assist pathologists in accelerating diagnostic turnaround through targeted slide review and, coupled with other clinical and pathological features, could improve the accuracy of identifying metastatic disease.

Following treatment, the prediction of long-term recurrence risk is crucial. In this respect, a multimodal DL prognostication tool dubbed Histopathology-based Endometrial Cancer Tailored Outcome Risk (HECTOR) has been developed [66]. This platform derives prognostic information from a combination of WSIs, image-based molecular class and anatomical stage, with lower HECTOR output scores being associated with more favourable prognostic markers (e.g., POLE mutant lesions and grade 1) and higher scores with poorer prognostic factors (e.g., oestrogen receptor negative and p53 mutant lesions). Since the model inputs are both accessible and widely used diagnostically, its clinical implementation looks promising.

## 5. Cervical Cancer

Cervical cancer ranks as the fourth commonest malignancy affecting women globally, with a global annual incidence of 660,000 cases in 2022 [67]. While developed countries have seen a decline or stabilisation in both the incidence and mortality rates attributed to cervical cancer over the past few decades [68,69], this trend contrasts starkly with the situation in low- and middle-income nations [67]. Disparities in screening, preventive measures (e.g., human papilloma virus (HPV) vaccination programmes) and socio-economic factors account for much of these inequalities. Indeed, cervical cancer is caused by infection from high-risk (hr) HPV subtypes (e.g., 16 and 18) and typically arises from precursor cervical intraepithelial neoplasia (CIN) or cervical glandular intraepithelial neoplasia (CGIN).

Screening plays an important role in early diagnosis of cervical cancer and its precursor lesions, including cervical cytology, hrHPV testing and DNA ploidy testing [70]. Although screening is efficiently practiced in North America and Europe, many countries in Asia and Africa are yet to achieve this goal [71]. In this regard, while cytology is a low-cost method and the most followed in developing countries, it has certain limitations, including a lower sensitivity in detecting precursor lesions compared to HPV testing [72,73]. Furthermore, the dearth of diagnostic pathologists in developing countries [74] poses a problem to its effective implementation. Fortunately, there are encouraging findings in using AI for screening purposes [75,76,77,78], with several studies performed in resource-limited settings [78,79,80,81,82]. From a research standpoint, DL/ML tools were found to have a higher specificity (>90%) for hrHPV serotypes and detecting high-grade atypia compared to detecting low-risk serotypes and low-grade atypia. As such, there is potential for complementary use of AI for screening, alongside conventional methods. As outlined above, HPV subtypes can be either high- or low-risk based on malignant potential: the former include subtypes 16, 18, 31, 33, 35, 45, 52 and 58, while the latter include subtypes 6, 11, 56, 59 and 66 [83]. Few studies have used AI models along with PCR assays on cytological specimens to differentiate the different HPV subtypes [78,84], which can support diagnoses. The integration of this approach with genomic profiles and biomarkers has enabled triaging methods [85] and risk stratification. The opportunities to automate cytology have been reviewed in detail [86] to reveal that, although the existing methods have increased the number of slides screened by cytopathologists, the accuracy by which results are interpreted decreases when large number of specimens are reviewed in a day. WSIs of histology and cytology specimens also present distinct challenges in computer vision due to differences between histology and cytology specimens. Histology WSIs include intact tissue architecture, showing organised structural layers that highlight spatial relationships among cells and tissues. By contrast, cytology WSIs contain dispersed cells, often isolated from their tissue context, which complicates spatial inference and orientation and can pose scanning challenges (and allied AI-based interpretation) typically owing to the multiple planes of focus. Nevertheless, advances in scanning technologies have overcome some of these issues, and Wang and colleagues developed a fully automated DL system to analyse cervical cytology specimen WSIs capable of detecting high-grade squamous intraepithelial lesions or squamous cell carcinoma with a precision of 0.93. As with other approaches, this requires further validation in a clinical setting but may provide a helpful adjunct screening tool for cervical cancer and its precursor lesions [87]. Another tool for automating the interpretation of cervical cytology is the Pap Smear Analysis Tool (PAT). The purported merit of this technology lies with its ability to screen out cytologically normal specimens (0% false negative rate), thereby enabling cytologists to concentrate on suspicious specimens and reducing both workloads and review times. Again, the potential of such platforms may come into their own in developing economies and healthcare systems where there is a paucity of both cytologists and funding [88]. Thus, further development into implementation of screening and diagnostic methods remains critical.

The incorporation of AI into histology has helped with the classification of cervical lesions. Pre-fed CNNs were able to distinguish malignant from non-malignant H&E-stained histological section WSIs of cervical biopsy specimens in the research setting [89], a stepping stone towards improving diagnostics. In this respect, Cheng and colleagues developed a tool that combined low- and high-resolution WSIs [90]. Images were first screened by a low-resolution model which located suspicious regions and generated location heatmaps. Areas with a probability greater than 0.5 were then cropped according to the heat maps and passed through high-resolution models to identify 10 lesional cells based on a probability score. Finally, a WSI classification model using a recurrent neural network (RNN) combined the features of these 10 lesional cells to determine the likelihood that the entire slide was positive for malignancy.

Akin to models that have been developed in other gynaecological malignancies, AI has made advances in terms of prognostication in cervical cancer too [91,92,93]. Therein, prediction models to stratify prognostic disease recurrence risk have been developed [91], formulated based on factors including age, tumour size, stromal invasion and adjuvant therapy, where they predicted disease-free survival and overall survival in post-surgery patients with early-stage cervical cancer. As highlighted above, these prediction models may have value in informing the development of future multimodal approaches incorporating the use of WSIs. Assigning a pathological risk score [93] with the help of information extracted by DL from WSIs has also helped personalise the risk of recurrence in individual patients.

## 6. Vulval and Vaginal Cancers

Vulval and vaginal cancers are rare gynaecological malignancies with around 47,336 and 18,819 new cases reported worldwide every year, respectively, in 2022 [94]. While squamous cell carcinoma (SCC) is the most prevalent histological type of vulval malignancy, others include basal cell carcinomas, malignant melanomas, vulvar Paget’s disease, verrucous carcinomas and adenocarcinomas. HPV is responsible for around 30–40% of SCCs, while the HPV-independent type can reportedly evolve on a background of chronic lichen sclerosis. Given that vaginal cancers arise most commonly from lesions in the vulva, cervix or other adjacent sites, primary vaginal cancer is defined as a disease with no history of adjacent lesions in the cervix and vulva. While these cancers may be HPV- (more commonly) or non-HPV-related, histological diagnosis following biopsy remains the diagnostic gold standard regardless.

The most significant work to date using image-based diagnostics has been a recent study exploring the use of CNNs in differentiating LSIL and HSIL from vaginal mucosa using colposcopic images (with a high specificity of 99.7%) [95]. Future AI techniques incorporating both macroscopic and microscopic images may provide a holistic approach combining the benefits of both point of care and subsequent histopathological validation. However, given the relative rarity of vulval and vaginal cancers, there has—unsurprisingly—been limited research in applying AI diagnostic and prognostic solutions in these pathologies. In part, this is a reflection of a combination of targeted funding to investigating other, more common cancers, as well as the relative scarcity of WSIs on which AI models can be trained and tested. However, adapting existing platforms applied to similar lesions with related aetiologies in other anatomical sites could direct future research endeavours. For example, AI has been used in oral SCCs from histopathological slides of biopsy specimens with a specificity of 0.92 [96]. Such methods could be translated to the vulval and vaginal SCC setting, where they could be applied to assist pathologists with diagnosis. Similarly, in malignant melanomas of the eyelid, DL algorithms have been developed to aid with diagnosis [97]. This approach could potentially be utilised to underpin the development of diagnostic algorithms of the rarer mucosal melanomas of the vulva. Thus, as AI research continues to evolve, potential advances made in other anatomical areas with comparable malignancies and their precursors could be adapted to the histopathological diagnosis of rare manifestations in other anatomical sites.

## 7. Discussion

**Figure 1:**
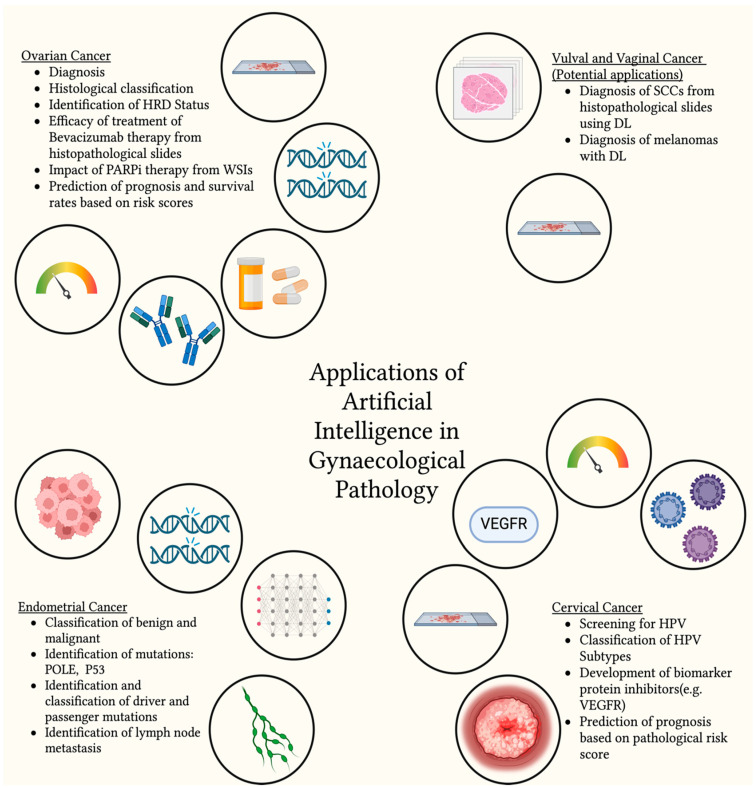
Existing applications of AI in the diagnostic setting for different gynaecological malignancies (Created in BioRender. Joshua, A. https://BioRender.com/w15v459, accessed on 21 January 2025).

The applications of AI in gynaecological malignancy histopathology could have transformative applications in the diagnosis, prognosis and management of these diseases, as highlighted by this review and summarised in Figure 1. The studies reviewed have been included in Table 1. However, many studies in the field remain dogged by several limitations which preclude their clinical use at present. Firstly, AI tools are frequently confined to the research environment rather than being properly trialled in a real-life clinical environment, which is a key contributor to establishing safety, efficacy and end-user (i.e., pathologist) engagement and adoption [42]. Secondly, most studies have a small sample size and can often use single-centre-source WSIs [78,85], which makes it difficult to accurately predict the comparability and universality of different AI model performances. Furthermore, technical challenges pertaining to platform agnosia can arise as a result of the use of scanners from different manufacturers that may impact algorithm performance because of differences in the optical and computed properties of digitised histopathology slides [98]. Differences in H&E staining protocols across histopathology laboratories could also introduce variability and affect consistency in analytical performance [99], although some of these hurdfles have been overcome by colour balance pre-processing. Technologies developed for resection samples may also encounter limitations when applied to biopsies or tissue samples with different histological contexts, such as lymph node metastases, due to their distinct architecture and morphology. In this regard, they may not represent the histopathological diversity encountered in the clinical setting [58] and fail to account for less common subtypes of malignancies [65]. For instance, while high-grade serous type in ovarian cancer and squamous cell carcinoma in cervical cancer are well represented, other, less common subtypes may be overlooked. Finally, studies aiming at developing clinically meaningful solutions should also pay particular attention to the nature of source specimens (e.g., frozen versus formalin-fixed only) [100], thus underscoring the value of multidisciplinary investigatory teams with input from pathologists, computer scientists, cancer biologists and biomedical scientists.

While there have been significant advances made in AI in the context of diagnostic pathology, current systems typically operate on completing a single target activity, such as morphological/molecular typing or immunohistochemical scoring, which can often be effectively standardised. By contrast, pathological diagnosis has increasingly become a multi-step process, often integrating multi-source data (e.g., histological subtyping/grading, immunohistochemistry and sequencing), where pathologists’ interpretation remains central. Moreover, the reality of working within the real-life variation in the quality of clinical specimens in terms of staining protocols, tissue fixation or artifacts (e.g., tissue folds, crushed cells and cell debris) means that any AI-based solution would have to be resilient to “non-standard” histology in WSIs and likely require continued pathologist overview to ensure diagnostic accuracy in any early adoption programme. Indeed, whilst AI-based solutions may prove to be valuable adjuncts with the potential to improve diagnostic service efficiency and cost, the threshold for replacing clinicians is unlikely to be met even in the presence of full automation, as pathologists will remain essential for rare lesion identification, diagnostic probability assessments, quality control and clinico-legal liability.

As a result, one of the areas requiring further investment is the development of meaningful datasets and image annotations (including those from different scanners to ensure platform agnosia). Since all forms of AI require input data such as images, text and genomic profiles, improvement in these spheres can further improve the performance of AI models, particularly with applications such as learning using privileged information (LUPI) [101]. Moreover, enhancing the quality of input data by incorporating diverse data sets with a variety of WSIs with representative variability in histological patterns from different populations can potentially eliminate the risk of bias arising in AI models [102]. In cases where there are few data sets available (e.g., lower genital tract malignancies), augmentation techniques can help refine data, especially those of histopathological images [103]. This has, for example, been explored in cervical cancer, with the development of synthetic images with real image similarity [104]. As such, further work in this area and expanding it to other gynaecological malignancies could contribute to further improving the functionality and performance of existing AI models.

The integration of AI models and tools into clinical practice faces multiple challenges, among which the issue of data drift features prominently. Data drift arises when AI models exhibit divergent performance in real-world environments compared to during their training phase. Alone, these changes are sufficient to invalidate the use of any model for use in a clinical setting. Incorporating explainability into AI models may facilitate addressing this challenge [105]. Explainability essentially refers to making the decisions and predictions made by AI algorithms more understandable and interpretable to humans and overcoming the so-called “black box” criticism often levelled at ML-based solutions. In this regard, dividing WSIs into functional units (based on cell structure and type) instead of spatial units (based on dimensions and pixel counts) could improve explainability upon pathology review. The use of manually curated features (i.e., quantitative features of size, colour and morphology) to classify images while using ML tools may also impact explainability [106], although additional limitations including the time taken and the use of specialists (whose time these solutions aim to spare) need to be considered. Additionally, periodic retraining—and subsequent locking—of these models could contribute to mitigating the effects of data drift.

While implementing these AI models in clinical settings, the training of pathologists in order to efficiently utilise these tools is also critical. In this respect, an end-user-based study [107] found that high usability, user involvement and levels of trust play a role in the reception and willingness to adopt AI in pathology. Active collaboration between the data scientists creating the technology and the pathologists using it is crucial, where adequate support from leadership, space, staffing, storage and scanners are important for effective integration of the two fields [108]. 

The final consideration on the path to clinical adoption is the clearance of regulatory hurdles. Various country-specific organisations (e.g., US FDA, UK National Institute for Clinical Excellence, etc.) evaluate such technologies from the standpoint of efficacy, reproducibility, safety, patient benefit and cost–benefit evaluations, which are not only critical to providing an independent review of these platforms but also in offering credibility and establishing reimbursement channels.

## 8. Conclusions

Thus, the untouched potential of AI in gynaecological malignancies can have promising applications in the clinical setting. However, the co-operation between data scientists and clinical pathologists with further input from regulatory bodies, healthcare system infrastructure managers and pharmaceutical companies will be critical to ensuring that these technologies are appropriately tailored to answering clinical diagnostic queries, thereby maximising their efficacy and applicability. There is undoubtedly a need for an overarching vision that can see beyond the short-term additional investment in the extra steps of digitising pathology services and implementing AI in the patient pathway that, in the long term, will yield time, accuracy and cost benefits for pathologists and, most importantly, their patients.

## Figures and Tables

**Table 1:** Study cohort details of AI-based analytical platforms applied to the gynaecological pathology diagnostic setting.

Paper Authors	Morphological Subtyping	Molecular Subtyping	Prognostication	Data Source	Training/Validation Test Set Size	External Validation
**Ovarian Cancer**						
Wu et al. [25]	No	No	No	First Affiliated Hospital of Xinjiang Medical University	Original-Training: 5914; Validation: 1478 Augmented-Training: 65,050; Validation: 16,262	No
BenTaieb et al. [27]	Yes	No	No	Unclear	Training set size: 73	No
Farahani et al. [28]	Yes	No	No	OVCARE Archives, University of Calgary	Training set size: 948 Validation test set size: 60	No
Bourgade et al. [32]	No	Yes—BRCA	No	University Hospitals of Nantes and Rennes, TCGA	Training set size: 1,040,149 tumour tiles Validation test set size: 111,727 tumour tiles	Yes
Shafi et al. [36]	No	Yes—HRD	No	Unclear	Training set size: 150	No
Wang et al. [37]	No	No	Yes	Tri-Service General Hospital and the National Defense Medical Center, Taipei, Taiwan	Training data set size: 187; Testing data set size: 101	No
Laury et al. [38]	Yes	No	No	HUS Helsinki University Hospital	Training set size: 205 Test set size: 22	No
Laury et al. [40]	Yes	Yes—JUN	Yes	Helsinki Biobank	Training set: 205; Validation set: 22	No
Yang et al. [41]	Yes	No	Yes	TCGA-OV, Prostate, Lung, Colorectal, and Ovarian Cancer Screening Trial (PLCO) and Harbin Medical University Cancer Hospital	2449 slides	Yes
Wu et al. [42]	No	Yes—HRD, BRCA	Yes	TCGA-OV	Training data set: 72 Test data set: 18	No
**Endometrial Cancer**						
Fell et al. [48]	No	No	No	NHS Greater Glasgow and Clyde Biorepository and Pathology Tissue Resource	Training data set: 1248 Validation data set: 616 Test data set: 863	No
Zhao et al. [49]	Yes	No	No	Unclear	Training data set: 6248; Validation data set: 1564; External validation data set: 1631	Yes
Sun et al. [50]	Yes	No	No	Third Affiliated Hospital of Zhengzhou University	Data set size: 3302; External validation data set: 200	Yes
Mohammadi et al. [52]	Yes	No	No	iCAIRD	Training data set: 998 Validation data set: 466 Test data set: 864	No
Goyal et al. [53]	Yes	No	No	Dartmouth Health, TCGA	Training data set: 929; Validation data set: 100	Yes
Fremond et al. [54]	No	Yes—POLE, p53abn, MMRd, NSMP	Yes	PORTEC-1, PORTEC-2, PORTEC-3, TCGA, TransPORTEC pilot study, Medisch Spectrum Twente cohort	Training set data size: 1240; Test set data size: 393	No
Hong et al. [58]	Yes	Yes (multiple)	Yes	TCGA, Clinical Proteomic Tumor Analysis Consortium, NYU Hospitals	Data set size: 496	Yes
Suzuki et al. [61]	No	No	Yes	Kyoto Cohort, ICI Cohort, TCGA	Data set size: 966	No
Feng et al. [65]	Yes	No	Yes	West China Second University Hospital, Qingdao University, Affiliated Yantai Yu Huang Ding Hospital, Beijing Maternal and Child Health Care Hospital	Internal data set size: 2104 External data set size: 533	Yes
Volinsky-Fremond et al. [66]	Yes	Yes	Yes	PORTEC 1,2,3, University Medical Center Groningen, Leiden University Medical Center	Test data set: 353; Training data set: 1408; External validation data set: 310	Yes
**Cervical Cancer**						
Holmström et al. [76]	Yes	No	No	Kinondo Kwetu Health Services Clinic, Kinondo, Kwale County	Training data set: 360; Validation data set: 361	No
Wong et al. [78]	Yes	No	No	Cervical Cytology Laboratory, Department of Pathology, The University of Hong Kong	Training data set: 485; Validation data set: 120	No
Bao et al. [80]	Yes	No	No	Hubei, China	Training data set: 103,793	No
Nakisige et al. [82]	Yes	No	No	Uganda Cancer Institute, International Agency for Research on Cancer, Leiden University Medical Center	Training data set: 70; Test data set: 20; Validation data set: 10	No
Pathania et al. [84]	Yes	No	No	Unclear	Training data set: 13,000	Yes
Tian et al. [85]	Yes	Yes	No	The First Affiliated Hospital of Sun Yat-sen University	30 Samples	No
Wang et al. [87]	Yes	No	No	Department of Pathology, Tri-Service General Hospital, National Defense Medical Center, Taipei, Taiwan	Training data set: 97; Test data set: 46	No
William et al. [88]	Yes	No	No	Mbarara Regional Referral Hospital	Dataset 1: 917; Dataset 2: 497; Dataset 3: 60	No
Cheng et al. [90]	Yes	No	No	Multiple hospitals	Training set size: 46,810; Test set size: 6617; Validation set size: 10,229	No
Chu et al. [91]	Yes	No	Yes	Qilu Hospital of Shandong University	Training data set: 385; Validation data set: 96	No
Obrzut et al. [92]	No	No	Yes	Department of Obstetrics and Gynaecology of the Rzeszow State Hospital in Poland	Unclear	No
Chen et al. [93]	No	No	Yes	Multiple hospitals	Training data set: 836; Validation data set: 354	No
Mascarenhas et al. [95]	Yes	No	No	Tertiary Care Centre (Centro Materno Infantil do Norte)	Training/Validation data sets: 51,525; Test data set size: 5725	No

## Data Availability

No new data were created or analyzed in this study. Data sharing is not applicable to this article.

## Abbreviations

The following abbreviations are used in this manuscript:

AI	Artificial Intelligence
WSI	Whole Slide Imaging
FDA	Food and Drug Administration
DL	Deep Learning
HRD	Homologous Recombinant Deficiency
PARPi	Poly-ADP-ribose polymerase inhibitor
CNN	Convolutional Neural Networks
EMA	European Medicines Agency
OCDPI	Ovarian Cancer Digital Pathology Index
POLE	Polymerase ε
MMRd	Mismatch Repair Deficient
NSMP	No Specific Molecular Type
WHO	World Health Organisation
CLAM	Clustering-constrained Attention-based Multiple instance learning
AUROC	Area Under the Receiver Operating Curve
PORTEC	Post Operative Radiation Therapy in Endometrial Cancer
TLS	Tertiary Lymphoid Structures
HECTOR	Histopathology based Endometrial Cancer Tailored Outcome Risk
MIL	Multiple Instance Learning
HPV	Human Papilloma Virus
CIN	Cervical Intraepithelial Neoplasia
CGIN	Cervical Glandular Intraepithelial Neoplasia
PAT	Pap Smear Analysis Tool
RNN	Recurrent Neural Network
PLCO	Prostate, Lung, Colorectal, and Ovarian Cancer Screening Trial
SCC	Squamous Cell Carcinoma
